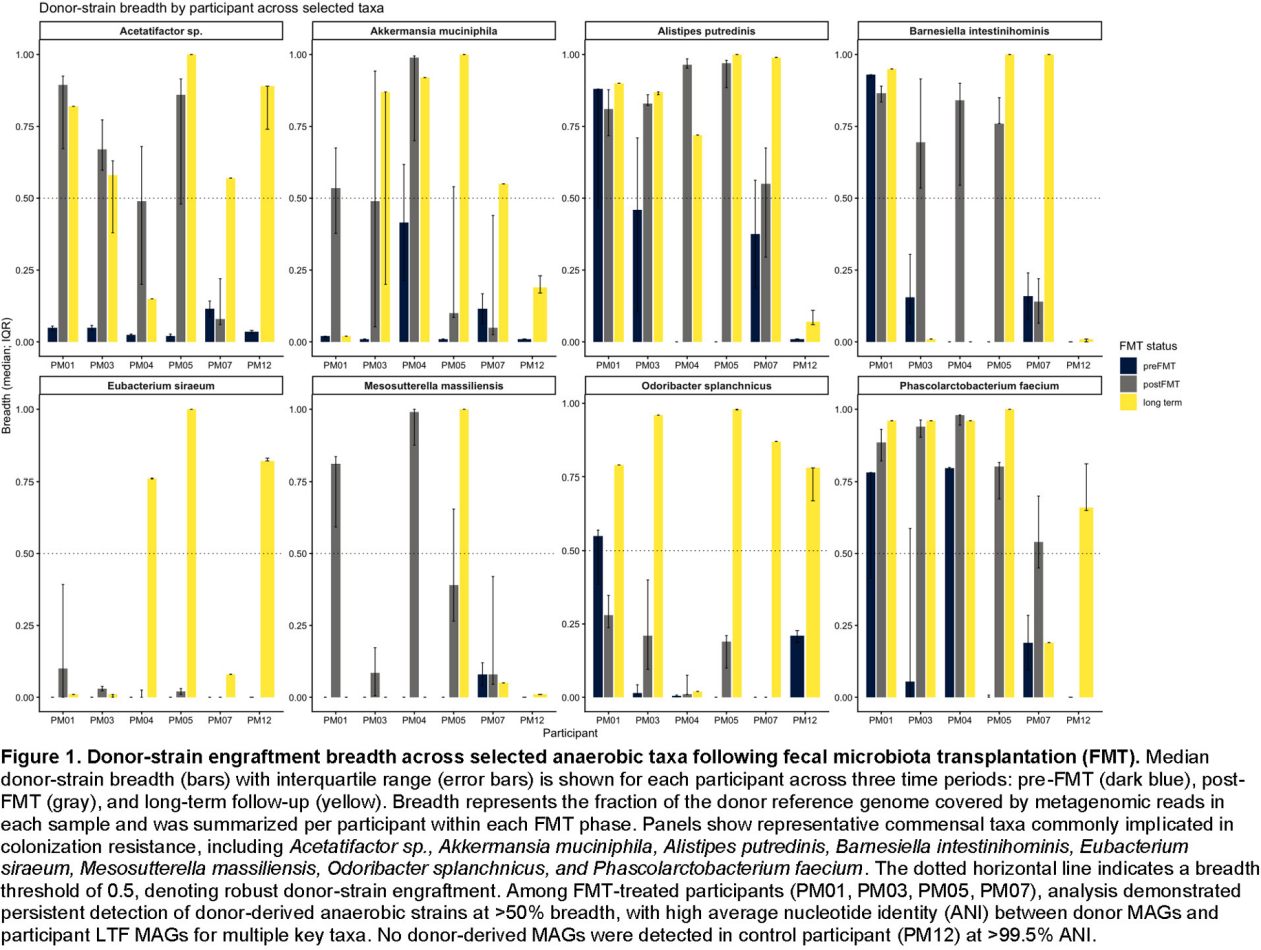# 32 Leadership Experiences of Infection Prevention and Control Professionals: LEAD-IP Study

**DOI:** 10.1017/ash.2026.10433

**Published:** 2026-06-23

**Authors:** Ahmed Babiker, Catherine Brink, Amanda Strudwick, Sarah Lohsen, Colleen Kraft, Sarah Satola, Konstantinos T Konstantinidis, Michael Woodworth

**Affiliations:** 1 Rush Medical Center; 2 Emory University; 3 Emory University Hospital; 4 Emory University School of Medicine; 5 Division of Infectious Diseases, Department of Medicine, Emory University, Atlanta, GA

## Abstract

**Background:** Microbiome restoration through fecal microbiota transplantation (FMT) is a promising decolonization strategy that reduces multidrug-resistant organism (MDRO) colonization and infection. However, the strains mediating MDRO decolonization remain poorly defined, and long-term follow-up (LTF) data after FMT for MDRO decolonization are limited. We enrolled participants from a completed randomized trial of FMT for MDRO decolonization (PREMIX, NCT02922816) into an LTF study to characterize long-term engraftment dynamics of donor-derived anaerobic taxa and their association with sustained MDRO decolonization. **Methods:** Participants provided stool samples every six months for up to five years following completion of the trial. Samples underwent selective culturing for MDROs using chromogenic agars, with organism identification and susceptibility confirmation by MALDI-TOF and Vitek2. MDRO isolates underwent whole-genome sequencing (WGS), and stool samples underwent metagenomic sequencing. To determine similarity between baseline and LTF MDROs, pairwise comparison of baseline and LTF MDRO isolates was performed with a unique strain defined as < 99.5% average nucleotide identify difference (ANI) to other strains. To determine engraftment of donor derived anaerobic taxa construction of metagenome-assembled genomes (MAG) from donor and participants samples was performed. Donor MAGs detected at <50% breadth in LTF metagenome samples were compared to corresponding species-level MAGs from participant LTF samples, with unique MAGs defined as <99.5% ANI like strains above Results Six PREMIX participants were enrolled to the LTF study, five of whom received FMT and one control. 80% (4/5) of FMT treated patients have remained decolonized (culture negative) while 0% (0/1) of control patients have remained decolonized at most recent follow up (median[IQR] 3.6 [3.4,4.0] years). The single FMT-treated participant with MDRO detection in LTF samples had evidence of a new acquisition compared to the baseline MDRO, whereas the MDRO detected in the control participant was identical to the baseline strain based on ANI cut offs. Among FMT-treated participants, metagenomic analysis demonstrated persistent detection of donor-derived anaerobic strains at <50% breadth, with high ANI between donor MAGs and participant LTF MAGs for multiple key taxa, including Acetatifactor sp., Akkermansia muciniphila, Alistipes putredinis, Barnesiella intestinihominis, Eubacterium siraeum, Mesosutterella massiliensis, Odoribacter splanchnicus, and Phascolarctobacterium faecium, consistent with long-term engraftment (Figure 1). No donor-derived MAGs were detected in control participants at <99.5% ANI. **Conclusions:** FMT was associated with long-term durable decolonization potentially mediated by the engraftment and persistence of key donor derived anaerobic strains. Our findings of persistence of discrete taxa highlights the potential for developing rationally constructed strain-based microbiome therapeutics for decolonization and infection prevention.

**Figure:**